# Voices unheard: Bridging language gaps, ensuring equity and inclusion of non‐native speakers in health research and clinical trials

**DOI:** 10.1002/nop2.70048

**Published:** 2024-09-23

**Authors:** Lemma N. Bulto

**Affiliations:** ^1^ Caring Futures Institute Flinders University Adelaide South Australia Australia

**Keywords:** exclusion criteria, health research, language

In a globalized world, the ability to communicate in different languages has never been more important, especially in the healthcare context. However, despite the need for inclusion and diversity in many health care settings, significant gaps remain regarding the inclusion of non‐native speakers and individuals with language difficulties in health service and clinical research. This violates the principles of equity and fairness. In the current literature, number of published studies stipulate exclusion of study participants based on language proficiency (Boyde et al., 2018; Egleston et al., 2015; Muthukumar et al., 2021). For instance, a significant number (18.98%) of international clinical trials in USA exclude participants who can't understand English or are not native speakers, and only 2.7% specifically mentioned translation of research materials to another language (Muthukumar et al., 2021).

The most prominent impacts of excluding non‐native speakers from health research is the increase in health disparities. Language difficulties can prevent individuals from accessing important health information, participating in preventive interventions, and receiving timely diagnosis and treatment (Pandey et al., 2021). This exclusion widen the existing health disparities, as policies and interventions developed from such incomplete findings often fail to address the unique health challenges faced by non‐native speakers such as immigrants (Anwar et al., 2023; Egleston et al., 2015; Muthukumar et al., 2021).

The exclusion of participants on the basis of language underestimates the rigour and generalizability of findings. Research that does not adequately represent the linguistic and sociocultural diversity of the population may overlook critical factors that affect health outcomes (Glickman et al., 2011). For example, socio‐cultural differences in health behaviours, genetic factors prevalent in certain ethnic groups, and varying responses to treatments can all be ignored in translation research findings. By ensuring that research participants are representative of the general population, research findings can be more precisely applied to real‐world context, thereby improving the effectiveness and acceptability of health intervention programs. From an ethical perspective, exclusion individuals with language difficulties is against the principles of justice and respect for human, which are basis to ethical research undertaking. All individuals should have the opportunity to contribute to and benefit from scientific findings, regardless of their social and economic backgrounds, and the language they speak.

For instance, within the sphere of cardiovascular disease (CVD) research, excluding individuals with language difficulty from participation in CVD prevention programs undermines the effectiveness and equity of health program. Non‐native speakers are often immigrant minorities with unique socioeconomic and cultural factors that affect their cardiovascular health (Almoussa & Mattei, 2023). Ignoring these groups can lead to incomplete intervention that doesn't meet their specific needs. This exclusion widens the existing health disparities, as non‐native speakers do not benefit from health the program, leading to poor health outcomes. Research and inclusive intervention strategies are needed to achieve effective cardiovascular prevention and treatment in all populations, improving overall health outcomes and reducing health disparities.

Addressing the problem of exclusion based on language in health research needs to be addressed through a multifaceted strategy (Egleston et al., 2015; Glickman et al., 2011; Murray & Buller, 2007). First, health programs and research studies should seek language support services, including interpretation and translation, to facilitate the involvement of non‐native speakers and individuals with language difficulty. This includes providing participant information sheet and informed consent in multiple languages and employing multilingual research staff.

Health professionals and researchers should receive training in cultural competency to better understand and address the needs of diverse populations. This training can help bridge communication gaps and enhance inclusive research approach. In addition, research proposals should be designed with inclusivity in mind, aiming to recruit participants from diverse linguistic and sociocultural backgrounds. This can be achieved through community engagement and partnerships with organizations that serve non‐native speakers, such as those working with immigrants and underrepresented populations.

Health policymakers and research funding institutions should advocate for regulations that mandate the inclusion of linguistic minorities in research and health programs. This may involve revising funding criteria to prioritize studies that demonstrate efforts to include diverse populations. For instance, the Australian Heart Foundation prioritize funding for research projects that address priority populations including those from culturally and linguistically diverse background. Furthermore, researchers should collect and report data on the linguistic diversity of their study populations. This can help to identify gaps in inclusion and drive further improvements in future research practices (Chen et al., 2022; Muthukumar et al., 2021).

In general, the exclusion of participants based on language proficiency in health research and programs is a critical issue that violates the principles of equality, equity, and ethics. By addressing language barriers and adopting more inclusive practices, researchers and policy makers can ensure that health policies and research reflect the diversity of societies and meet the needs of the wide population. It is imperative that researcher and health policy makers move towards a more inclusive and equitable healthcare system where language is no longer a barrier to participation and health program access.

## Data Availability

No original data used.
